# Patient-reported gradual worsening reveals progression beyond MS subtypes

**DOI:** 10.1007/s00415-026-13648-w

**Published:** 2026-02-07

**Authors:** Jie Guo, Tomas Olsson, Lars Alfredsson, Anna Karin Hedström

**Affiliations:** 1 Department of Clinical Neuroscience, Center for Molecular Medicine, L8, Karolinska Institutet, 171 76 Stockholm, Sweden; 2 Department of Nutrition and Health, China Agricultural University, Beijing, China; 3 Institute of Environmental Medicine, Karolinska Institutet, Stockholm, Sweden; 4 Centre for Occupational and Environmental Medicine, Region Stockholm, Stockholm, Sweden

**Keywords:** Multiple sclerosis, Phenotype, Subtype classification, Progression

## Abstract

**Background:**

Gradual worsening in relapsing–remitting MS (RRMS) may precede the formal transition to secondary progressive MS (SPMS). We aimed to quantify self-reported gradual worsening, assess concordance with clinically recorded subtype, and identify baseline predictors of discordance.

**Methods:**

We included 1640 participants with incident RRMS from a population-based study (2005–2019). A 2021 follow-up survey captured patient-reported gradual worsening. Clinical data, including Expanded Disability Status Scale (EDSS) scores and SPMS classification, were obtained from the Swedish MS registry. Discordance with clinically recorded subtype was modeled using logistic regression stratified by subtype. Time to EDSS 3 and EDSS 4 were summarized with Kaplan–Meier estimates within subtype by self-reported worsening, and secondary Cox proportional hazards models were fitted stratified by subtype with self-reported worsening as the exposure.

**Results:**

Among participants classified as RRMS, 24% reported gradual worsening, while 23% of those classified as SPMS did not. Kaplan–Meier curves showed clear within-subtype separation by self-reported worsening, consistent with higher hazards of reaching EDSS 3 and EDSS 4 among those reporting worsening. In RRMS, older age and higher baseline EDSS were associated with self-reported gradual worsening despite RRMS classification. In SPMS, self-reported worsening preceded clinical classification by 4.1 years.

**Conclusions:**

Patient-reported gradual worsening aligns with disability accumulation and may help identify progression earlier than subtype reclassification, supporting integration of structured patient reports into routine monitoring.

**Supplementary Information:**

The online version contains supplementary material available at 10.1007/s00415-026-13648-w.

## Introduction

The classification of multiple sclerosis (MS) into relapsing–remitting (RRMS), secondary progressive (SPMS), and other subtypes has long guided clinical decision-making and research design. However, accumulating evidence suggests that these phenotypic categories may oversimplify the disease course which often unfolds as a gradual and heterogeneous continuum [1]. Many individuals with RRMS eventually experience steady neurological worsening independent of relapses, a process now referred to as progression independent of relapse activity (PIRA) [2, 3]. Yet, the transition from RRMS to SPMS is typically recognized only retrospectively, often following a prolonged period of unacknowledged decline [4–6].

Defining SPMS remains a clinical challenge, with wide variation in how and when the transition is recorded across settings. Formal reclassification in clinical practice or national registries may lag behind symptom onset, particularly in the absence of clear relapse events or dramatic shifts in disability scores [4–6]. This lag can result in under-detection of early progression, delayed treatment adjustments, and misclassification in observational studies. Meanwhile, efforts to develop more sensitive tools for detecting progression, including composite scores, digital biomarkers, and imaging, remain in development or have limited applicability outside clinical trials [7–9].

Patient-reported outcomes offer a complementary perspective on disease progression. Gradual functional decline may be perceived by patients well before it is captured in clinical assessments. The clinical relevance of earlier recognition is becoming increasingly important by ongoing therapeutic studies targeting progressive mechanisms [10, 11]. Identifying early signs of worsening through patient-reported outcomes could support timelier monitoring and clinical decision-making. However, the extent to which subjective reports align with clinically recorded subtype classification remains unclear.

Accordingly, we used nationwide clinical and self-reported data to examine the alignment between MS subtype classification and self-reported gradual worsening, assess rates of discordance, and describe clinical and demographic characteristics. These analyses are intended to contribute to ongoing efforts to refine MS phenotyping and improve early detection of disease progression in both clinical and research contexts.

## Methods

We used data from participants in the Epidemiologic Investigation of Multiple Sclerosis (EIMS), a nationwide case–control study of incident MS in Sweden. Between April 2005 and December 2019, a total of 3567 individuals with newly diagnosed MS were recruited through hospital-based neurology departments, including all university hospitals. All cases met the McDonald criteria for MS [12, 13] as diagnosed by the treating neurologist, and completed a standardized baseline questionnaire on demographics, lifestyle habits, and environmental exposures. The case response rate was 93%. Further details on the study design have been described elsewhere [14].

**Fig. 1:**
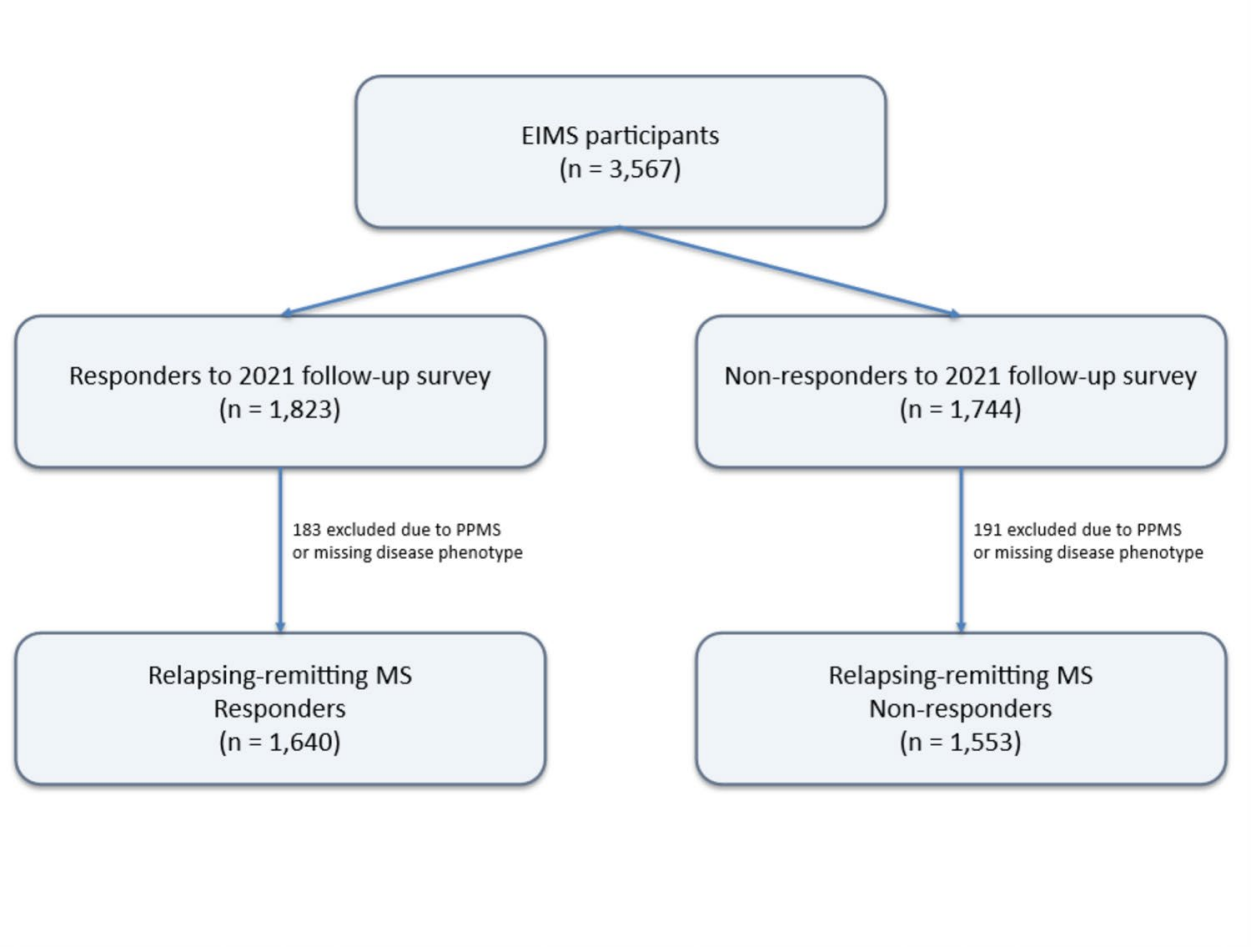
Study flow and analytic samples

In 2021, EIMS participants were invited to complete one digital follow-up survey that included questions on lifestyle changes, perceived health status, and MS-related symptoms, including the Multiple Sclerosis Impact Scale (MSIS)-29 [15]. A total of 1823 individuals responded. We excluded those with primary progressive MS (PPMS) (n = 74) and those with missing information on disease phenotype (n = 109), yielding a final sample of 1640 patients for analysis. A study flow diagram summarizing accrual, exclusions, and analysis sets is provided in Fig. 1.

Participants were linked to the Swedish MS register [16], a nationwide quality register where treating neurologists routinely report detailed clinical information on disease course, therapy, and functioning, in accordance with national guidelines. The register provided the clinically recorded MS subtype (RRMS/SPMS) as assigned by the treating neurologist during follow-up, longitudinal EDSS scores, and treatment history. Registry data were extracted with follow-up updated through 2021.

Self-reported gradual worsening was assessed in the 2021 questionnaire with a single global question. Participants indicated whether they experienced slow, steady progression over time, and if so, the calendar year when it began. Baseline predictors were obtained from the baseline questionnaire at or near the diagnostic visit.

Ethical approval was obtained from the Regional Ethical Review Board at Karolinska Institutet. The study was conducted in accordance with the Declaration of Helsinki and its subsequent amendments. All participants provided written consent.

### Statistical analysis

Descriptive statistics were used to compare clinical, demographic, and lifestyle characteristics across four groups defined by clinically recorded MS subtype (RRMS or SPMS) and self-reported gradual worsening (yes or no). Continuous variables were summarized using means and standard deviations (SD), and categorical variables using frequencies and percentages. Among RRMS participants who endorsed gradual worsening, we calculated the interval between the self-reported year of worsening onset and the 2021 follow-up survey and summarized the distribution using mean, median and interquartile range (IQR).

To investigate predictors of discordance between clinically recorded MS subtype and self-reported progression, we fitted separate logistic regression models stratified by subtype, coding discordance at the 2021 survey as reported worsening in RRMS and no reported worsening in SPMS. Candidate predictors measured at or near diagnosis included age, sex, disease duration, EDSS, smoking (current vs non-smoking), BMI (< 25, 25–30, or > 30 kg/m^2^), physical activity (low, moderate, moderate-high, or high), educational level (pre-secondary, secondary, or post-secondary education), ancestry (Nordic vs non-Nordic), and disease-modifying (DMT) initiation within 12 months of diagnosis to preserve temporal ordering. All models were additionally adjusted for calendar period of diagnosis (2005–2009, 2010–2014, 2015–2019) and years from diagnosis to 2021. Final models retained age, sex, EDSS at diagnosis, disease duration at diagnosis, and DMT exposure a priori. Other covariates were retained if statistically significant. We report odds ratios (ORs) and 95% confidence intervals (CI). For disability accumulation, we identified the first date with EDSS ≥ 3 and EDSS ≥ 4. Follow-up was administratively censored at 15 years from diagnosis or last registry entry. Kaplan–Meier estimates of time from diagnosis to EDSS milestones were stratified by clinically recorded subtype and self-reported worsening, restricted to participants with baseline EDSS < 3. We report the estimated probability of reaching each threshold at 5 and 10 years with 95% CI. Among SPMS participants who reported progression, we also calculated the difference between the recorded year of SPMS onset and the self-reported year of gradual worsening.

In secondary time-to-event analyses, we fitted Cox proportional hazards models for time to EDSS 3 and 4, stratified by clinically recorded subtype, with self-reported gradual worsening as the exposure. Analyses were restricted to participants with EDSS < 3 and adjusted for age at diagnosis, sex, disease duration at diagnosis, baseline EDSS, calendar period of diagnosis, years from diagnosis to 2021, and DMT initiation within 12 months. We report hazards ratios with 95% CIs.

To assess potential response bias, we assessed the comparability between respondents and non-respondents to the 2021 follow-up survey, by comparing baseline characteristics (age at disease onset, age at diagnosis, disease duration at baseline, sex, Nordic ancestry, baseline EDSS, and DMT exposure) using descriptive statistics and standardized mean differences (SMDs). All analyses were performed using SAS version 9.4 (SAS Institute Inc., Cary, NC), and visualizations were generated using Python (Matplotlib).

## Results

Participant characteristics at diagnosis and follow-up in 2021 varied across groups defined by register-based MS subtype and self-reported gradual worsening (Table 1). Among participants classified as RRMS, nearly one in four (n = 325/1383) reported gradual worsening, despite lacking a SPMS diagnosis. At diagnosis, these individuals had higher EDSS and greater symptom burden, compared to those without self-reported progression. Conversely, 59 individuals (23%) classified as SPMS did not report gradual worsening. By 2021, participants with self-reported gradual worsening had more pronounced disability and symptom burden than those without, both among those classified as RRMS and SPMS. Among RRMS participants who reported gradual worsening (n = 325), the last registered relapse occurred on average 5.5 years before the reported onset of worsening (SD 5.1). The self-reported onset occurred a mean of 6.1 years before the 2021 survey (median 5, IQR 3–9), corresponding to a mean age at onset of progression of 44.1 years (SD 10.8).

**Table 1:** Characteristics of participants by clinically recorded MS type and self-reported gradual worsening

Register-based MS type	RRMS		SPMS	
Self-reported worsening	No progression	Progression	No progression	Progression
N	1058	325	59	198
*Characteristics at diagnosis (*± *1 year)*				
Age (SD)	35.5 (9.6)	38.8 (10.3)	47.1 (10.5)	46.2 (10.0)
Female, n (%)	780 (73.7)	238 (73.2)	37 (62.7)	135 (68.2)
Nordic, n (%)	870 (82.2)	255 (78.5)	55 (93.2)	170 (85.9)
Disease duration (SD)	2.7 (4.7)	3.3 (4.9)	5.6 (7.2)	6.6 (8.2)
EDSS (SD)	1.4 (1.2)	1.8 (1.3)	2.6 (1.7)	2.7 (1.5)
MSIS phys (SD)	13.6 (16.8)	24.8 (21.4)	27.9 (23.3)	36.2 (21.4)
MSIS psych (SD)	24.3 (21.8)	35.7 (23.6)	29.6 (18.7)	37.1 (24.7)
Smoking, n (%)	185 (17.5)	64 (19.7)	13 (22.0)	39 (19.7)
BMI (SD)	24.8 (4.8)	25.3 (5.4)	25.2 (4.0)	25.5 (4.4)
Physical activity, n (%)	459 (43.4)	107 (32.9)	23 (39.0)	58 (29.3)
*Treatment up to 2021*				
Never DMT, n (%)	56 (5.3)	16 (4.9)	6 (10.2)	28 (14.1)
Only platform DMT, n (%)	350 (33.1)	100 (30.8)	38 (64.4)	88 (44.4)
Only high-efficacy DMT, n (%)	481 (45.5)	166 (51.1)	9 (15.3)	61 (30.8)
Escalation, n (%)^a^	171 (16.2)	43 (13.2)	6 (10.2)	21 (10.6)
*Characteristics in 2021 (*± *1 year)*				
Age (SD)	47.1 (10.0)	50.6 (10.3)	60.1 (10.5)	59.0 (9.8)
Disease duration (SD)	14.2 (6.0)	15.1 (6.0)	18.7 (8.5)	19.3 (8.7)
EDSS (SD)	1.5 (1.3)	2.7 (1.5)	3.7 (1.9)	5.2 (1.7)
MSIS phys (SD)	36.1 (14.3)	56.7 (18.1)	48.5 (18.3)	70.8 (18.6)
MSIS psych (SD)	43.6 (18.0)	62.4 (20.5)	47.9 (19.4)	61.5 (21.1)
Cognitive functioning (SD)^b^	1.6 (0.7)	2.2 (0.8)	1.9 (1.0)	2.1 (0.9)
Smoking, n (%)	101 (9.6)	30 (9.2)	8 (13.6)	19 (9.6)
BMI (SD)	25.7 (4.9)	26.2 (5.6)	26.3 (5.0)	26.3 (4.9)
Physical activity, n (%)	569 (53.8)	104 (32.0)	20 (33.9)	41 (20.7)

*RRMS* relapsing–remitting multiple sclerosis; *SPMS* secondary progressive multiple sclerosis; *EDSS* expanded disability status scale; *MSIS* multiple sclerosis impact scale; *BMI* body mass index; *DMT* disease-modifying therapy; *SD* standard deviation

^a^initial platform therapy followed by high-efficacy DMT

^b^self-reported cognitive functioning was assessed on a four-level scale, ranging from no or minimal difficulties to severe, disabling difficulties, with higher scores indicating greater impairment

In logistic regression models restricted to individuals with clinically recorded RRMS, older age at diagnosis (OR 1.04, 95% CI 1.02–1.05) and higher baseline EDSS (OR 1.21, 95% CI 1.10–1.34) were associated with increased odds of self-reported gradual worsening (Table 2). Among individuals classified as SPMS, no significant associations were observed for age, sex, disease duration, baseline EDSS, or DMT exposure. Across subtypes, none of the other prespecified covariates, including smoking, BMI, physical activity, educational level, or Nordic ancestry, were significantly associated with discordance. We did not observe sex-specific differences in the relationship between subtype classification and patient-reported gradual worsening.

**Table 2:** Predictors of discordance between clinically recorded MS subtype and self-reported gradual worsening

RRMS (n = 1383)	
Age	**1.03 (1.02–1.05)**
Male	1.0 (reference)
Female	1.07 (0.79–1.45)
Disease duration	1.02 (0.99–1.04)
EDSS at diagnosis	**1.20 (1.09–1.33)**
DMT exposure	2.22 (0.83–6.17)
SPMS (n = 257)	
Age	1.02 (0.99–1.06)
Male	1.0 (reference)
Female	0.74 (0.38–1.42)
Disease duration	0.99 (0.94–1.03)
EDSS at diagnosis	0.94 (0.76–1.16)
DMT exposure	1.06 (0.26–4.28)

Logistic regression models run separately for participants with register-based RRMS and SPMS. In the RRMS group, the outcome was self-reported gradual worsening. In the SPMS group, the outcome was absence of self-reported gradual worsening, i.e., disagreement with the SPMS classification. All models were additionally adjusted for calendar period of diagnosis and years from diagnosis to 2021

*RRMS* relapsing–remitting multiple sclerosis; *SPMS* secondary progressive multiple sclerosis; *EDSS* expanded disability status scale; *DMT* disease-modifying therapy

**Fig. 2:**
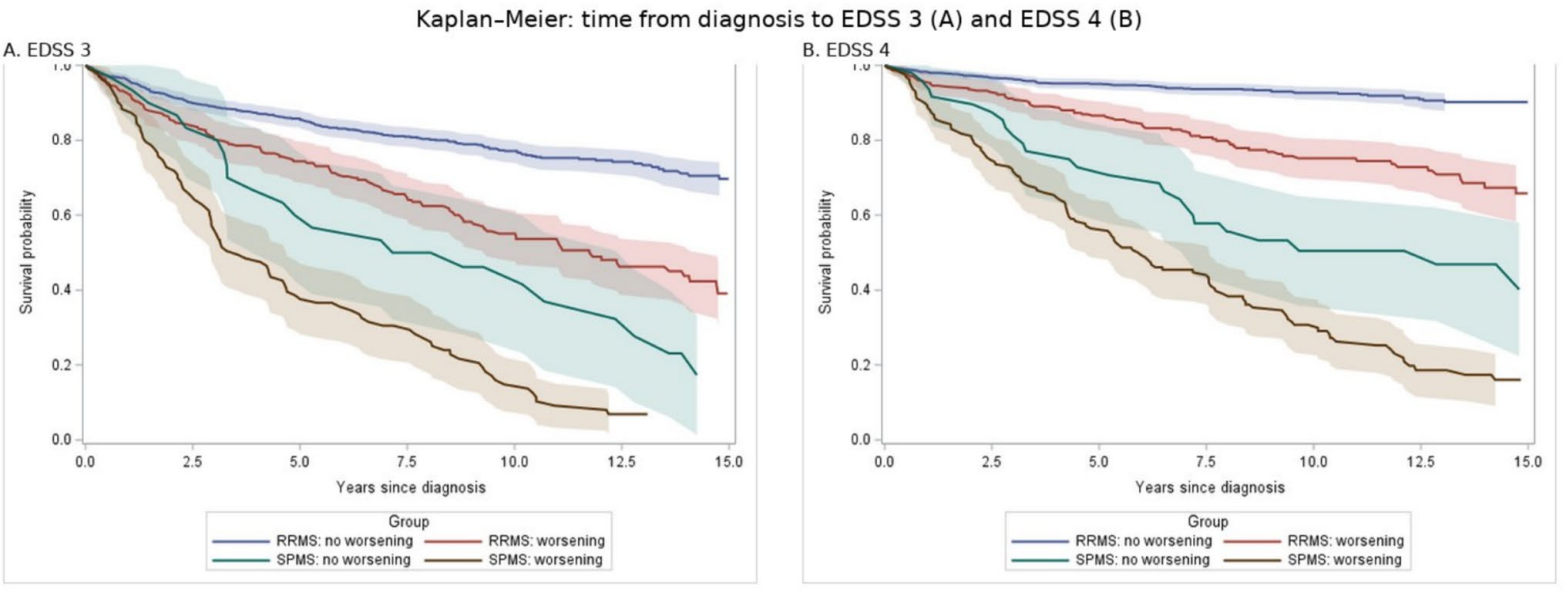
Kaplan–Meier curves for time from diagnosis to EDSS milestones by clinically recorded subtype and self-reported worsening, truncated at 15 years. RRMS = relapsing–remitting multiple sclerosis, SPMS = secondary progressive multiple sclerosis, EDSS = expanded disability status scale. Panel A shows EDSS 3 and Panel B shows EDSS 4. Shaded bands indicate 95% confidence intervals

Kaplan–Meier estimates showed clear within-subtype gradients by self-reported worsening (Fig. 2). The estimated probability of reaching EDSS 3 and EDSS 4 at 5 and 10 years is summarized in Table 3. Among SPMS participants who reported gradual worsening, the mean discrepancy between the self-reported and clinically recorded year of SPMS onset was 4.1 years (SD 4.2). Cox models stratified by clinically recorded subtype yielded consistent associations, with higher HRs among those reporting worsening (eTable 1).

**Table 3:** Five- and 10-year estimated probabilities of EDSS 3 and EDSS 4 by clinically recorded subtype and self-reported worsening

Group	EDSS 3		EDSS 4	
	5 years (%)	10 years (%)	5 years (%)	10 years (%)
RRMS, no reported worsening	14	23	5	7
RRMS, reported worsening	26	45	13	25
SPMS, no reported worsening	40	54	27	50
SPMS, reported worsening	61	85	44	69

*RRMS* relapsing–remitting multiple sclerosis; *SPMS* secondary progressive multiple sclerosis; *EDSS* expanded disability status scale

Respondents and non-respondents to the 2021 follow-up survey were largely comparable at baseline, with modest differences for age at diagnosis (ASMD 0.15) and Nordic ancestry (ASMD 0.18) (eTable 2).

## Discussion

In our cohort, nearly one quarter of individuals classified as RRMS reported gradual worsening, whereas a similar proportion of those classified as SPMS did not. Kaplan–Meier and Cox analyses showed clear within-subtype separation, with higher probabilities and hazards of reaching EDSS 3 and 4 among participants who reported worsening. Among SPMS, self-reported worsening preceded registry-based subtype reclassification by an average of 4.1 years.

At a group level, MS subtype classification remained clinically informative, with higher rates of worsening and disability accumulation among individuals classified as SPMS compared with RRMS. At the same time, the observed discordance indicates that patient-reported gradual worsening captures clinically relevant information not fully reflected in registry-based subtype assignments, consistent with MS progression as a gradual and heterogeneous process that may be under-recognized in routine care and incompletely captured by clinician-assigned subtypes.

The clinical relevance of identifying early progression has increased with the emergence of therapies targeting progressive disease mechanisms [10, 11]. Although results from such trials remain mixed, the ability to identify individuals at risk of progression earlier in the disease course may become increasingly important for monitoring strategies and treatment decisions. In this context, patient-reported gradual worsening represents a pragmatic and patient-centered signal that may complement existing clinical assessments during periods when progression is not yet formally recognized. We observed no clear association between early DMT initiation and discordance between patient-reported worsening and recorded subtype. This finding should be interpreted cautiously. Confounding by indication is likely, as individuals perceived to be at higher risk of progression may have been more likely to initiate therapy early. In addition, currently available DMTs primarily target inflammatory disease activity and may have limited impact on gradual progression. The wide confidence intervals observed further suggest limited precision rather than absence of effect.

Accumulating evidence suggests that neurodegeneration begins early and proceeds gradually, even in patients classified as RRMS [17]. The observed average delay of 4 years between self-reported worsening and SPMS designation in our cohort further illustrates this classification lag. This misalignment reflects an ongoing debate whether phenotypic subtypes such as RRMS and SPMS should be retained or replaced by a continuum-based disease model [18–20]. While subtype labels facilitate structured care and trial design, they risk oversimplifying the disease course. A hybrid model that retains practical subtype categories while integrating longitudinal patient-reported data, imaging [21, 22], and biomarkers [7–9], may better capture the continuous and dynamic nature of MS progression. Such an approach could improve both clinical decision-making and the precision of research on progressive disease, while also providing a framework for future studies to address mechanistic questions regarding the overlap or divergence of pathological processes across RRMS and SPMS.

Several limitations should be considered. The 2021 response rate was modest, introducing the possibility of non-response or attrition bias. Respondents and non-respondents were broadly similar at diagnosis, which reduces but does not eliminate the risk of selection bias. Although self-reported gradual worsening was retrospective and is inherently subjective and may be influenced by mood or expectations, its alignment with objective outcomes supports its validity as a complementary source of information. The reported onset of worsening was typically remote from inflammatory disease activity. Among RRMS participants reporting worsening, the last registered relapse occurred on average more than 5 years before the reported onset, supporting relapse-independent processes.

The observed lag between patient-reported gradual worsening and registry-based transition to SPMS is also likely to reflect limitations of the EDSS. EDSS is predominantly driven by ambulation and captures non-motor symptoms poorly, despite their substantial impact on daily functioning. Self-reported cognitive difficulties were more pronounced among participants reporting gradual worsening, supporting that patient-reported worsening encompasses domains not well captured by EDSS. Subtle changes in motor performance may likewise influence perceived disability without immediately affecting EDSS scores. Although SPMS classification is not formally determined by EDSS alone, EDSS trajectories and milestones play a central role in informing subtype reclassification. Our findings therefore likely reflect both the limited sensitivity of EDSS to early or non-motor progression and the inherent delay in recognizing sustained progression in clinical practice.

Analyses stratified by SPMS status may have reduced precision within strata. We adjusted for calendar year and time from baseline to 2021 to address variable follow-up, yet residual time related confounding may remain. Baseline DMT exposure was defined as initiation within 12 months of diagnosis to preserve temporal ordering, but residual confounding by indication cannot be excluded. EDSS milestones were defined from the first registry entry with EDSS 3 or 4, without a fixed confirmation window, because registry visits occur at variable intervals in routine care. As a result, short-term fluctuations could shift the recorded milestone date and, in some cases, misclassify transient changes as events. However, this is likely to introduce nondifferential misclassification, which would tend to attenuate between-group differences rather than create them.

In conclusion, patient-reported gradual worsening captures clinically relevant information beyond subtype labels. Incorporating patient-reported outcomes alongside EDSS may help identify early progression and refine MS phenotyping in both practice and research.

## Supplementary Information

Below is the link to the electronic supplementary material.

Supplementary file1 (DOCX 19 KB)

## Data Availability

Anonymized data underlying this article will be shared on reasonable request from any qualified investigator who wants to analyze questions that are related to the published article.
