# Supplementary material for: Patient-reported gradual worsening reveals progression beyond MS subtypes
Source: J Neurol. 2026 Feb 7;273(2):117. doi: 10.1007/s00415-026-13648-w (PMC12881162; doi:10.1007/s00415-026-13648-w)
Supplement: Supplementary file 1 — Supplementary file1 (DOCX 19 KB) [file 415_2026_13648_MOESM1_ESM.docx]

eTable 1. Cox models for time to EDSS 3 and 4, by clinically recorded subtype (RRMS and SPMS). HRs for reported worsening vs no reported worsening

| RRMS | | | | | |
| --- | --- | --- | --- | --- | --- |
|  | N | Time | EDSS 3 (%) | HR (95% CI) | HR (95% CI) |
| No reported worsening | 925 | 8.7 (4.6) | 207 (22.4) | 1.0 (reference) | 1.0 (reference) |
| Reported worsening | 257 | 7.8 (4.9) | 119 (46.3) | 2.27 (1.82-2.86) | 2.17 (1.72-2.70) |
|  | N | Time | EDSS 4 (%) | HR (95% CI) | HR (95% CI) |
| No reported worsening | 925 | 9.9 (4.3) | 58 (6.3) | 1.0 (reference) | 1.0 (reference) |
| Reported worsening | 257 | 9.8 (4.4) | 50 (19.5) | 3.57 (2.63-5.00) | 3.23 (2.33-4.55) |
| SPMS | | | | | |
|  | N | Time | EDSS 3 (%) | HR (95% CI) | HR (95% CI) |
| No reported worsening | 32 | 8.0 (5.6) | 23 (71.9) | 1.0 (reference) | 1.0 (reference) |
| Reported worsening | 103 | 5.1 (4.5) | 94 (91.3) | 2.00 (1.25-3.23) | 1.85 (1.16-3.03) |
|  | N | Time | EDSS 4 (%) | HR (95% CI) | HR (95% CI) |
| No reported worsening | 32 | 10.8 (5.5) | 11 (34.4) | 1.0 (reference) | 1.0 (reference) |
| Reported worsening | 103 | 7.8 (4.9) | 74 (78.8) | 2.00 (1.30-3.03) | 1.92 (1.25-3.03) |

RRMS=relapsing-remitting multiple sclerosis, SPMS=secondary progressive multiple sclerosis, EDSS=expanded disability status scale; HR=hazard ratio; CI=confidence interval.

eTable 2. Baseline characteristics of EIMS participants by response to the 2021 follow-up survey.

| Total | Respondents | Non-respondents | P value | ASMD |
| --- | --- | --- | --- | --- |
| N | 1640 | 1553 |  |  |
| At age onset (SD) | 34.5 (10.1) | 33.3 (10.4) | 0.23 | 0.12 |
| Age at diagnosis (SD) | 37.9 (10.56) | 36.2 (11.05) | 0.08 | 0.15 |
| Disease duration (SD) | 3.3 (5.53) | 2.9 (5.07) | <0.01 | 0.08 |
| Nordic, n (%) | 1350 (82.3) | 1161 (74.8) | <0.01 | 0.18 |
| Female, n (%) | 1190 (72.6) | 1097 (70.6) | 0.39 | 0.04 |
| Baseline EDSS (SD) | 1.7 (1.4) | 1.7 (1.4) | 0.64 | 0.02 |
| DMT, n (%) | 1592 (97.1) | 1497 (96.4) | 0.28 | 0.04 |

EIMS=epidemiological investigation of multiple sclerosis; ASMD=absolute standardized mean difference. Values <0.10 are typically interpreted as negligible, 0.10–0.20 as small imbalance.
